# Restoring Sperm Quality Post-Cryopreservation Using Mitochondrial-Targeted Compounds

**DOI:** 10.3390/antiox11091808

**Published:** 2022-09-14

**Authors:** Macarena Gonzalez, Tanisha Prashar, Haley Connaughton, Michael Barry, Rebecca Robker, Ryan Rose

**Affiliations:** 1 Robinson Research Institute, School of Biomedicine, The University of Adelaide, Adelaide 5000, Australia; 2 Fertility SA, St. Andrew’s Hospital, Adelaide 5000, Australia

**Keywords:** cryopreservation, semen, antioxidants, mitochondria, reactive oxygen species, DNA damage, spermatozoa

## Abstract

While critical for male fertility preservation, cryopreservation damage reduces sperm quality and fertilization potential. This study investigated whether the addition of mitochondrial-targeted, antioxidant compounds, also known as Mitochondrial activators, to the cryopreservation medium could protect sperm quality during cryopreservation. For this, semen samples from men undergoing IVF/ICSI treatment, which were donated for research, underwent cryopreservation in the absence or presence of BGP-15, MitoQ and L-carnitine. Fresh semen and thawed sperm samples from the same participant were analyzed for indicators of sperm quality: sperm viability, kinetics, mitochondrial reactive oxygen species (ROS) levels, Mitochondrial Membrane Potential (MMP) and DNA damage. Cryopreservation significantly reduced sperm viability and motility and predicted mucous penetration. BGP-15, MitoQ and L-carnitine improved sperm motility, whilst the addition of L-Carnitine prevented the loss of sperm viability during cryopreservation. Both BGP-15 and L-carnitine reduced sperm DNA oxidative damage, but only BGP-15 significantly reduced DNA fragmentation. More importantly, BGP-15 increased sperm predictive mucous penetration and MMP and reduced DNA oxidation. Our results show that the addition of BGP-15 or L-carnitine to the cryopreservation medium improves sperm quality post-thawing, highlighting the potential of mitochondrial antioxidants to improve long-term fertility preservation in males.

## 1. Introduction

Sperm cryopreservation is the conservation of sperm at extremely low temperatures of about −196 °C [1]. It is used to preserve sperm samples for later use, such as that of men undergoing toxic radiation therapy or suffering from malignant diseases, for storage in donor banks and to manage assisted reproductive technology (ART) cycles for couples who are physically distant [2]. While important for fertility preservation, cryopreservation induces damage to the sperm, including altered DNA integrity, DNA fragmentation, modified metabolic function, mitochondrial injury, cell dehydration and acrosome damage (reviewed in [3]). While oocyte and embryo freezing have been repeatedly refined to increase successful outcomes during ART, sperm cryopreservation techniques have changed little since their development in the 1950s [4].

The production of high ROS levels by the cryopreservation process damages the sperm cell, altering sperm reproductive potential and reducing fertilization [5]. Furthermore, the ROS imbalance induces a loss of viability, mitochondrial dysfunction and DNA damage, among other things [6,7]. Mitochondrial dysfunction results in lipid peroxidation and a reduction in Mitochondrial Membrane Potential (MMP) [8], a predictor of sperm’s fertilization capacity [9,10]. The damage to sperm due to high oxidative stress negatively affects ART success and is related to low pregnancy rates [11], especially after intra-uterine insemination [11,12]. Therefore, there is a need for interventions to reduce cryopreservation damage and improve sperm quality after thawing.

Oxidative stress in various tissues is countered by antioxidants and antioxidant enzymes, which reduce the risk of damage by excessive oxidation [13]. Mature sperm possess low levels of cytoplasmic antioxidants [14], more acutely lowered by the loss of enzymatic activities of the peroxidation defence enzyme and superoxide dismutase in cryopreserved human sperm [15]. Therefore, the provision of exogenous antioxidants to protect sperm against oxidative stress during cryopreservation and thawing has been extensively evaluated (reviewed in [16]). Historically, the addition of non-enzymatic antioxidants in a sperm cryopreservation medium has increased motility parameters [17,18,19], protected acrosome integrity [20,21,22,23], reduced lipid peroxidation [17,24,25], increased MMP [26,27,28] and increased fertilization potential [19,29]. Mitochondrial activators—or, therapeutic agents that directly target the mitochondria—are an attractive additive for sperm media because of their antioxidant capacity as well as their mitochondria activation and reviving properties. One such compound that improves sperm quality is L-carnitine or β-hydroxy-γ-N-trimethylaminobutyric acid, a derivative of the amino-acid lysine. L-carnitine supplementation during human sperm cryopreservation improved sperm motility and vitality [30,31]. Another such compound is mitoquinone (MitoQ), a CoQ10 derivative which is a lipophilic cation combined with the antioxidant component quinone [32]. The supplementation of MitoQ in a sperm vitrification medium, a rapid freezing method, decreased oxidative stress, reducing sperm membrane and DNA damage [33]. Both L-carnitine and MitoQ have been used to protect mammalian sperm during cryopreservation, yet it is still not clear which one is more successful in protecting sperm quality. Furthermore, BGP-15 [(O-[3-piperidino-2-hydroxy-1-propyl]-nicotinic amidoxime)] is a nicotinic amidoxime derivate and heat shock protein activator, initially developed to treat insulin resistance [34], which has been shown to reduce mitochondrial ROS production in cell culture models and oocytes, as well as to increase MMP in oocytes when added to an in vitro maturation culture medium [35,36]. Despite demonstrating a diverse range of cytoprotective effects, the mechanism by which BGP-15 exerts its function is still unknown.

Therefore, we explored the impact of mitochondrial activators—BGP-15, MitoQ and L-Carnitine—during sperm cryopreservation on the preservation of sperm quality and compared their individual effects to those of fresh and standard cryopreservation (control) samples from the same participant’s semen. We assessed sperm motility and kinetics, viability, Predictive Mucous Penetration (PMP), mitochondrial superoxide levels and MMP and sperm DNA damage (fragmentation and oxidation) as measures of sperm health post-thawing. We hypothesized that the addition of these mitochondrial activators in the cryopreservation media before freezing will preserve sperm quality.

## 2. Materials and Methods

Reagents were acquired from Sigma-Aldrich, St. Louis, MO, USA, unless otherwise indicated.

### 2.1. Semen Collection

Semen samples were donated to a biobank by patients undergoing in vitro fertilisation/intracytoplasmic sperm injection (IVF/ICSI) treatments at an Adelaide-based ART provider, Fertility SA, and were deemed excess to treatment. St. Andrew’s Hospital Human Research Ethics Committee (HREC) approved the collection and storage of these samples (STAND HREC Project #93). The use of samples in this proposal was approved by The University of Adelaide HREC (#H-2021-084).

This study was approved by St Andrews HREC and The University of Adelaide HREC via a consent waiver as an alternative to consent, as it uses otherwise discarded material and is of low/negligible risk. Therefore, no consent form was needed.

### 2.2. Semen Cryopreservation

Semen from 34 patients (Table 1) were divided into five equal aliquots (0.5 mL semen in each), with one aliquot analysed fresh as a non-cryopreserved control. Other aliquots were mixed dropwise over a 30 s period at a 1:1 semen-to-treatment ratio: Control (Quinn’s Advantage Sperm Freeze cryoprotectant, SAGE-In Vitro Fertilisation, Cooperssurgical Inc., Målov, Denmark), BGP-15 (Control + 20 µM BGP-15 (Molcore Biopharmatech Co., Ltd., Hangzhou, China)), MitoQ, (Control + 1 µM MitoQ (provided by MitoQ Ltd., Auckland, New Zealand)) and L-carnitine (Control + 7.4 mM L-carnitine). As semen was diluted with the treatment, the final concentrations for each pharmaceutical treatment were: BGP-15 10 µM, MitoQ 500 nM and L-Carnitine 3.7 mM. L-Carnitine concentrations were determined by previous literature [37,38], whilst BGP-15 and MitoQ concentrations were chosen because they improved sperm motility and vitality whilst reducing DNA damage in sperm from humans and mice, respectively (unpublished observations). Sterile water (Pfizer Inc., New York, NY, USA) was used as the vehicle to dissolve BGP-15 and L-Carnitine. Additionally, 100% Ethanol was used to dissolve MitoQ powder, followed by sterile water for further dilution. The volume of the vehicle added for each treatment was negligible; therefore, sterile water was not added to the control cryoprotecant. Groups were respectively prioritised depending on the volume of semen donated. Mixed semen samples were filled and sealed into 0.5 mL cryogenic straws (Cryo Bio System, L’Aigle, France) that were placed in a −80 °C freezer for 20–24 h, and then transferred to liquid nitrogen (−196 °C), where the samples were stored for a minimum of 10 weeks to a maximum of 20 weeks. Therefore, each sample was exposed to treatment during preparation (mixing and loading), freezing, cryostorage and thawing.

### 2.3. Sperm Motility and Kinetic Analysis

Prior to analysis, cryopreserved semen samples were removed from liquid nitrogen and thawed for 10 min at room temperature before analysis. Fresh and thawed cryopreserved samples were diluted 1:1 with Biggers, Whitten & Whittingham (BWW) sperm media [39] at 37 °C. Sperm motility was analysed by a Sperm Class Analyser: Computer-Aided Semen Analysis (CASA) System (MICROPTIC, Barcelona, Spain). A total of 3 µL of semen solution was transferred into a 20-micron glass disposable counting chamber (Birr BioSciences BV, Vreeland, The Netherlands) for motility analysis using the SCA motility analysis software (MICROPTIC) by recording a minimum of 500 sperm cells. The CASA system provided a detailed analysis of sperm motility and various kinetic parameters including curvilinear velocity (VCL), straight-line velocity (VSL), average path velocity (VAP), the linearity of forward progression (LIN: ratio of VSL to VCL), path straightness (STR: ratio of VSL/VAP) and path wobble (WOB: ratio of VSL/VAP). These kinetics measures are used to calculate the predicted mucous penetration (PMP) or the percentage of sperm cells with the ability to penetrate cervical mucous. These are sperm cells that have a VAP ≥ 25 µm/s and an STR ≥ 80% [40].

### 2.4. Sperm Mitochondrial Analysis

The LIVE/DEAD Fixable Far Red dead cell stain kit (Invitrogen by Thermo Fisher Scientific, Waltham, MA, USA) was used to exclude dead sperm cells which appear fluorescent with excitation/emission at ~633/655 nm. Sperm viability was calculated as the percentage of live (or dye-negative) cells from the total number of sperm cells.

MitoSox Red (MSR) mitochondrial superoxide stain (Invitrogen) was used to detect mitochondrial reactive oxygen species (ROS) in live sperm cells at an emission/excitation of 510/580 nm. Then, 0.4 M MSR, 0.1 M LIVE/DEAD stain solutions in BWW media were added to the semen samples and incubated at 37 °C for 15 min.

The JC-1 Mitochondrial Membrane Potential (MMP) Probe (Invitrogen) was used to detect the membrane voltage in live sperm cells, discriminating between high (red), medium (orange) and low (green) MMP sperm at an emission maximum of ~590 nm. Then, 0.27 M JC-1, 0.1 M LIVE/DEAD stain solutions in BWW media were added to the semen samples and incubated at 37 °C for 15 min.

All samples were centrifuged at 500× *g* for 5 min after incubation, reconstituted in BWW and then transferred to 5 mL round-bottom polystyrene tubes (Corning Falcon, Glendale, AZ, USA). Both were assessed using the FACS-Canto II Flow cytometer (Becton Dickinson, Franklin Lakes, NJ, USA), recording a total of 10,000 events per sample and processing the resulting data outputs using FlowJo software (Becton Dickinson).

### 2.5. Sperm DNA Damage Analysis

To identify DNA oxidation by detecting 8-hydroxy-2′-deoxyguanosine (8-OHdG), a marker of DNA oxidation [41], sperm cells from both fresh and thawed samples were centrifuged at 500× *g*, and the pellet was resubstituted in a de-condensation buffer containing 2 mM DL-Dithiothreitol and 0.5% Triton X-100 for 10 min at room temperature. The cells were then fixed in 4% paraformaldehyde for 15 min at 4 °C. After washing with Phosphate buffered saline (PBS) solution, the cells were blocked using 1.5% goat serum and then probed with 1:50 DNA/RNA antibody (Novus Biologicals, Littleton, CO, USA) in PBS solution at 4 °C overnight. The cells were washed again and probed with 1:400 Alexa Flour 488, goat anti-mouse IgG secondary antibody (Invitrogen) in PBS. Imaging was performed using an Olympus IX81 inverted fluorescent microscope (Olympus Corporation, Shinjuku, Tokyo, Japan) under an excitation/emission of 490/525 nm for the immunodetection of 8OHdG stain, counting a total of 100 sperm.

The sperm chromatin structure (HALO) assay was used to detect sperm DNA fragmentation. Semen aliquots were stored at −80 °C prior to analysis, thawed at room temperature for 10 min, mixed with 1% agarose, smeared on a 0.65% agarose pre-coated slide and kept at 4 °C until set. The slides were then covered with 0.08 N HCl for 7 min for the acid-denaturation of sperm DNA, and then both the neutralising and lysing solutions were applied consecutively for 10 and 5 min, respectively, and prepared as previously described [42]. The slides were then washed with MilliQ water and dehydrated with 70%, 90% and 100% ethanol for 2 min each, followed by DAPI stain (Invitrogen). Imaging was conducted using an inverted fluorescent microscope (Olympus IX81) with an excitation/emission of 350/470 nm for the detection of sperm DNA, counting a total of 100 cells.

### 2.6. Statistical Analysis

Statistical analysis was performed using GraphPad Prism version 9.0 for Windows (GraphPad Software, La Jolla, CA, USA). The effects of cryopreservation and mitochondrial activator treatment within individual patient samples were analysed by a repeated measures mixed model with post-hoc pairwise comparisons using a paired, two-tailed Student’s *t*-test. Comparisons were drawn between all cryopreserved groups and fresh samples, and then each mitochondrial activator treatment was compared to the control. Statistical significance was determined at *p* < 0.05.

## 3. Results

### 3.1. The Addition of Antioxidants to a Cryopreservation Medium Improves Sperm Motility Post-Cryopreservation

**Figure 1:**
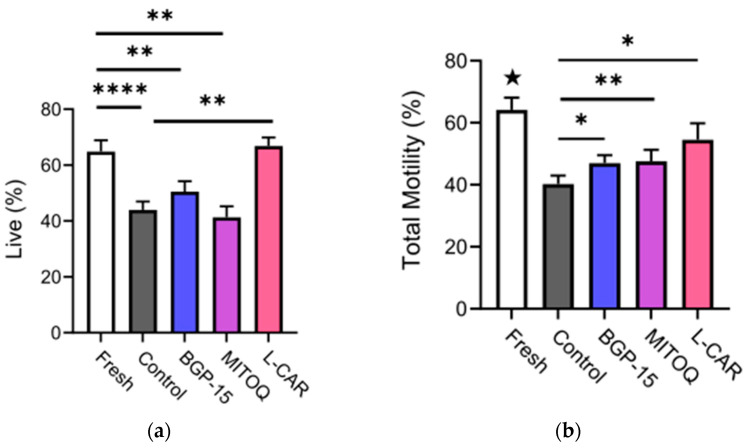

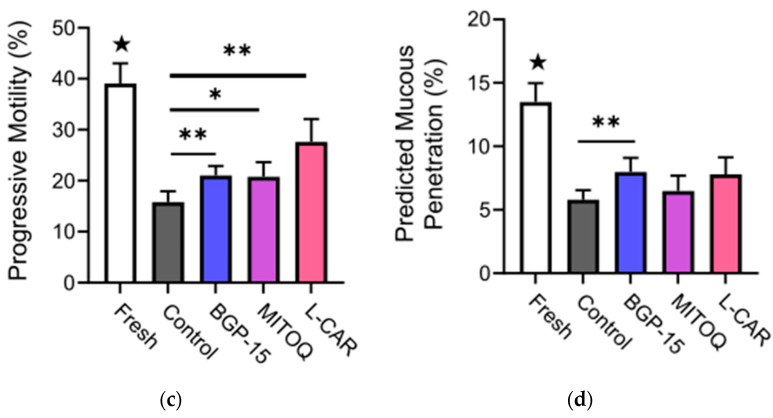
**Effect of mitochondrial activators on sperm motility and kinetics**. (**a**) Percentage of live sperm in all pre- (fresh) and post-cryopreservation samples; (**b**) Percentage of motile sperm from the total; (**c**) Percentage of progressive motile sperm from the total; (**d**) Predicted mucous penetration for motile sperm populations. Fresh (n = 34), control (n = 34), BGP-15 (n = 34), MitoQ (n = 22), L-car (n = 11). Data shown as mean ± SEM. Statistical analysis was the repeated measures mixed model with post-hoc pairwise comparisons. ★ indicates that all cryopreserved groups are significantly different from the fresh group; * *p* < 0.05, ** *p* < 0.01, **** *p* < 0.0001.

The cryopreservation of fresh semen samples without and with the addition of mitochondrial activators affected cell viability (*p* < 0.0001) (Figure 1a), which was reduced by 33.8% in the frozen control (*p* < 0.0001), by 23.3% in the BGP-15-treated group (*p* = 0.01) and by 36.3% in the MitoQ-treated group (*p* = 0.003) compared to the fresh samples (Figure 1a). However, the within-patient comparisons showed a 35.8% increase in sperm viability in the L-carnitine group compared to the control (*p* = 0.0004), which was similar to that of the fresh samples (Figure 1a). Cryopreservation also reduced the sperm total (*p* < 0.0001) and progressive motilities (*p* < 0.0001) (Figure 1b,c, respectively). These were reduced in all cryopreserved samples compared to the fresh samples (★ *p* < 0.05). The total motility of the semen cryopreserved with BGP-15 (*p* = 0.02), MitoQ (*p* = 0.05) and L-carnitine (*p* = 0.009) was increased by 14.3%, 15.5% and 26.2%, respectively, compared to the frozen control (Figure 1b). Similarly, all mitochondrial activator treatments increased progressive motility by 24.9% (*p* = 0.005), 24% (*p* = 0.04) and 42.8% (*p* = 0.009), respectively, compared to the control (Figure 1c).

The sperm kinetics were overall decreased by cryopreservation; however, the STR for the medium motile sperm and the VSL, VAP, LIN and WOB for the rapid motile sperm were preserved when mitochondrial activators were used compared to the control (Table 2). Predictive mucous penetration was reduced in all cryopreserved groups compared to the fresh samples for the motile sperm population (★ *p* < 0.05) (Figure 1d). However, BGP-15 treatment increased the predictive mucous penetration by 27.5% for the motile sperm population (*p* = 0.005) compared to the control (Figure 1d).

### 3.2. Semen Cryopreservation Induces Sperm mtROS

**Figure 2:**
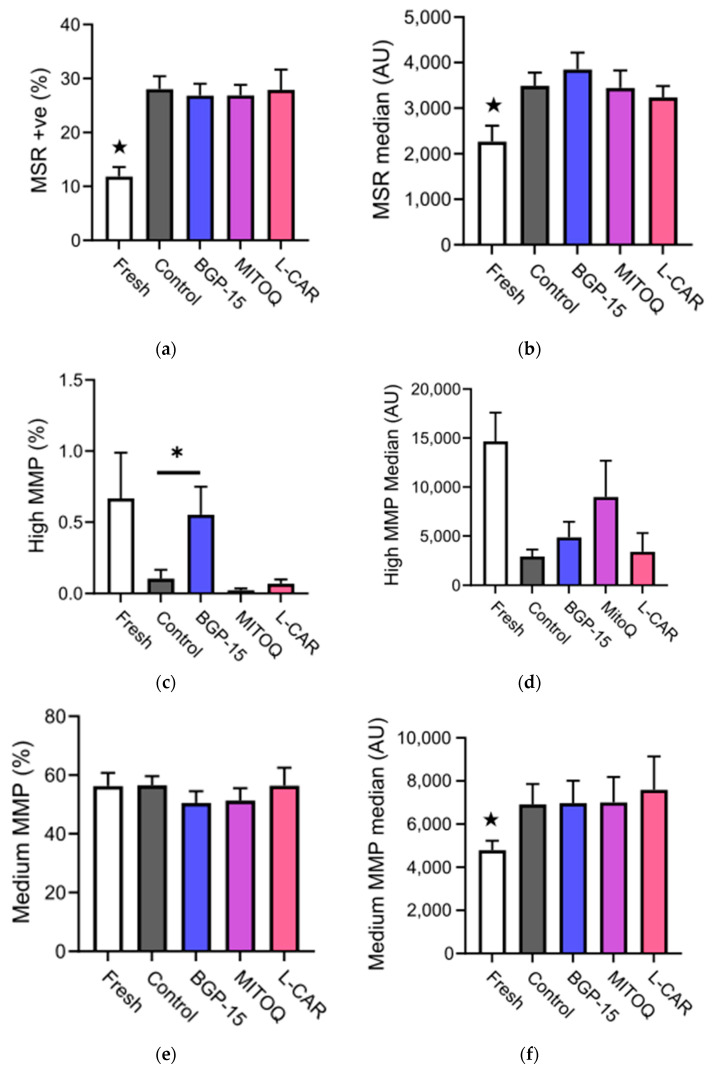
**Effect of mitochondrial activators on sperm superoxide levels and mitochondrial membrane potential.** (**a**) Percentage of sperm with high mitochondrial ROS in all pre- (fresh) and post-cryopreservation samples, as measured by MitoSox Red mtROS stain; (**b**) Average fluorescence intensity (median) of MitoSox Red mtROS stain per sperm cell; (**c**) Percentage of sperm with high Mitochondrial Membrane Potential (MMP), as measured by JC-1 MMP stain; (**d**) Average fluorescence intensity (median) of JC-1 MMP stain in sperm cells with high MMP; (**e**) Percentage of sperm with medium MMP; (**f**) Average fluorescence intensity (median) of JC-1 MMP stain in sperm cells with medium MMP. Fresh (n = 32), control (n = 32), BGP-15 (n = 32), MitoQ (n = 22), L-Car (n = 9). Data shown as mean ± SEM. Statistical analysis was the repeated measures mixed model with post-hoc pairwise comparisons. ★ indicates that all cryopreserved groups are significantly different from the fresh group; * *p* < 0.05.

Cryopreservation increased the percentage of live sperm cells with high mitochondrial ROS levels (*p* < 0.0001) and those with high ROS fluorescence intensity (*p* < 0.0001) (Figure 2a,b, respectively). Both ROS measures were greater post-cryopreservation compared to the fresh samples (★ *p* < 0.05) (Figure 2a,b). Sperm cells with a high MMP were increased by 81.4% in the BGP-15-supplemented samples compared to the control (*p* = 0.04), which was similar to the levels found in the fresh samples (Figure 2c), although there was not a significant effect of cryopreservation on the fluorescence intensity in this population (Figure 2d). While there was no effect of cryopreservation on the percentage of sperm with a medium MMP (Figure 2e), this population had higher fluorescence intensity in all cryopreserved groups compared to the fresh samples (★ *p* < 0.05) (Figure 2f).

### 3.3. Mitochondrial Activators Reduced Sperm DNA Damage Caused by Freezing

**Figure 3:**
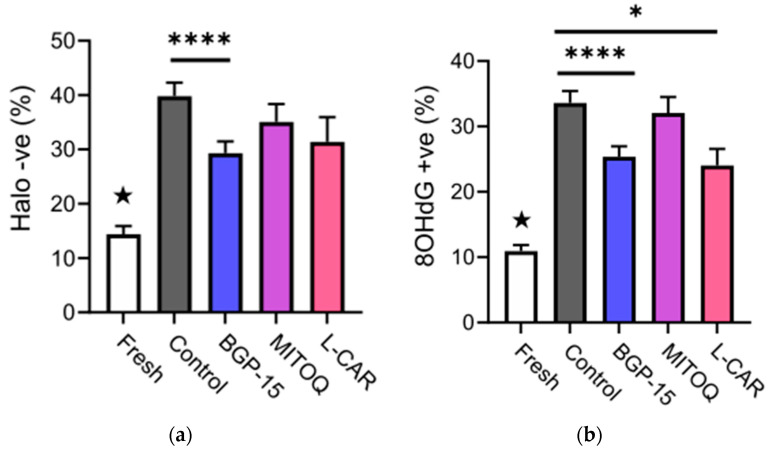
**Effect of mitochondrial activators on sperm DNA damage markers.** (**a**) Percentage of sperm with DNA fragmentation for pre- (fresh) and post-cryopreservation; (**b**) Percentage of sperm with DNA oxidative damage. Fresh (n = 34), control (n = 34), BGP-15 (n = 34), MitoQ (n = 22), L-car (n = 11). Data shown as mean ± SEM. Statistical analysis was the repeated measures mixed model with post-hoc pairwise comparisons. ★ indicates that all cryopreserved groups are significantly different from the fresh group; * *p* < 0.05, **** *p* < 0.0001.

Cryopreservation produced a strong increase in sperm DNA fragmentation (*p* < 0.0001) and oxidation (*p* < 0.0001, F = 44.5) (Figure 3a,b, respectively). Both DNA damage markers were higher in all cryopreserved samples compared to the fresh samples (★ *p* < 0.05). However, only the BGP-15-treated samples presented significantly reduced sperm DNA fragmentation compared to the frozen control (26.4%; *p* < 0.0001). In comparison, DNA fragmentation was 34% in the MitoQ group and 30.7% in the L-carnitine group, compared to 39.8% in the control (*p* = 0.07 and *p* = 0.06, respectively) (Figure 3a). Similarly, sperm DNA oxidation was also lowered by 23.8% in the BGP-15 group (*p* < 0.0001) and by 30.1% in the L-carnitine-supplemented group (*p* = 0.03) compared to the control (Figure 3b).

## 4. Discussion

**Figure 4:**
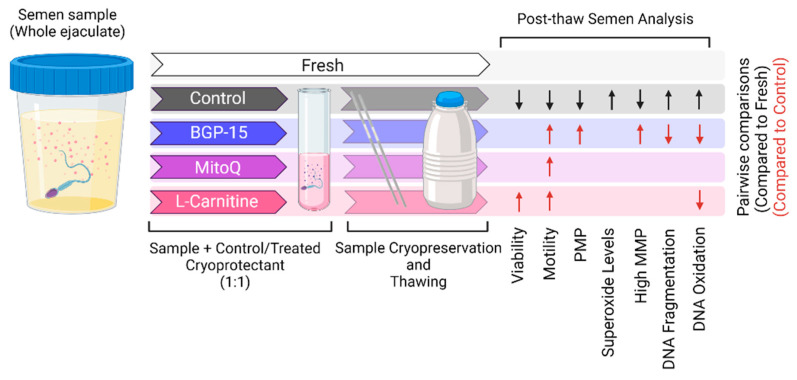
**Graphical summary of the cryoprotective effects of mitochondrial activators in human sperm cryopreservation.** Direct comparison of mitochondrial activators—BGP-15, MitoQ and L-Carnitine—to semen cryopreservation medium on thawed sperm quality. Arrows shown in the post-thaw semen analysis indicate significant (*p* < 0.05) increases or decreases compared to the fresh samples (black arrows) or compared to the control (red arrows).

There is an increased demand for sperm cryopreservation to preserve male fertility and for use in ART. The results of this study highlight the negative impacts of semen cryopreservation on sperm health, with increased sperm DNA fragmentation, DNA oxidative damage and mitochondrial ROS levels, in accordance with previous literature [5,6,43]. We and others have thus focused on finding ways to fix these defects by mitigating oxidative stress and increasing ATP through novel cryoprotective supplements, antioxidant supplementation, antifreeze proteins and laser irradiation [44,45,46,47,48] We show that mitochondrial activators can provide protection against the harmful effects of semen cryopreservation on sperm. We directly compared the use of BGP-15, MitoQ and L-carnitine in semen cryopreservation to fresh and standard cryopreservation (control) from the same ejaculate, with each compound demonstrating varied cryoprotective effects (Figure 4). BGP-15 was shown to effectively increase sperm motility, PMP and the percentage of high MMP sperm and decrease both DNA fragmentation and oxidative damage compared to the cryopreservation control. This is the first study demonstrating the beneficial effects of BGP-15 on human sperm, and, to our knowledge, this is the first time it has been used in the cryopreservation of human tissue.

During semen cryopreservation, sperm must endure several stressors, including ice formation, chemical toxicity and oxidative stress [49]. However, the loss of sperm function and health is likely due to cryopreservation-induced mitochondrial dysfunction. Sperm cryopreservation may result in Ca^2+^ overload from the prolonged opening of mitochondrial permeability transition pores, leading to ROS and Ca^2+^ release, deficient MMP, the depletion of ATP stores and the release of Cytochrome C [5]. As with all sperm cryopreservation methods, our human sperm freezing protocol reduced sperm motility and increased DNA fragmentation and oxidation, outcomes that are linked to lower fertilisation rates and poor-quality embryos [50,51].

Several groups have attempted to improve their semen cryopreservation system using mitochondrial activators. One of the most well characterised mitochondrial-targeted antioxidants, MitoQ, has been used extensively in non-human models. In human sperm, the addition of MitoQ to a cryopreservation medium improved sperm motility and lowered ROS and malondialdehyde concentration [52], while in vitrified sperm, it increased sperm motility, viability, acrosome membrane integrity and MMP and reduced DNA damage [33]. Although we observed that MitoQ was able to increase sperm motility compared to the control, we found no impact on sperm viability, ROS concentrations or DNA oxidative damage. This is in accordance with another recent report that MitoQ supplementation at concentrations higher than 0.2 µM showed no benefit to vitrified sperm [33]. Additionally, our freezing method, cryoprotectant and incubation times were different from those used in both previous studies using MitoQ treatment for human semen cryopreservation, and the difference in the results observed could thus be multifactorial.

L-Carnitine-supplementation has also been widely studied in semen freezing for species of agricultural interest; however, its application to the clinical ART space has been limited. Importantly, recent evidence shows that the treatment of semen with L-Carnitine prior to cryopreservation reduced sperm cryodamage in both asthenozoospermic [31] and normozoospermic men [30,31]. In agreement with these studies, we demonstrated that L-Carnitine improved sperm post-thaw motility and viability compared to the control. Furthermore, we saw an overall decrease in DNA damage with L-carnitine supplementation compared to the control. These findings are in contrast to one study which found no effect of L-carnitine supplementation on sperm DNA oxidation levels post-thaw [30], but they are similar to another which reported reduced sperm DNA fragmentation in L-carnitine-treated samples, albeit only in those from asthenoszoospermic men [31].

MitoQ’s antioxidant function stems from inhibiting mitochondrial ROS and supporting ATP production, mitigating cellular oxidative stress (reviewed in [53]), while L-Carnitine increases the transport of fatty acids to the mitochondria or β-oxidation, improving mitochondrial function and reducing ROS levels [54,55,56,57]. The molecular target of BGP-15 is unknown; however, there is clear evidence that it affects mitochondrial bioenergetics [35,36,58,59,60]. BGP-15’s exact mechanism of action in sperm is currently unknown and under investigation. It is unlikely that BGP-15’s effects are isolated to its ability to reduce the mitochondrial production of ROS [35], as no reduction in sperm superoxide levels were seen in this study. We demonstrated that the addition of the novel mitochondrial activator BGP-15 to the semen cryopreservation medium resulted in higher sperm motility, increased predictive mucous penetration, more sperm with a high MMP and reduced DNA damage, thus indicating that BGP-15 treatment protects sperm from cryodamage and improves its quality after thawing. Future studies should address the effect of BGP-15 treatment on sperm competence and fertilisation potential.

To make this study as clinically relevant as possible, all patients with excess semen donated for research purposes were included, independent of their age, BMI, smoking habits, alcohol consumption or infertility diagnosis. Thus, this group of patients adequately describes the patient population found at this clinic and fits our goal to improve cryopreservation outcomes for all patients, not a select subgroup. We considered that splitting patients up based on the mentioned demographic characteristics would reduce our sample size in each group, thus reducing statistical power. Further, DNA damage has been linked to age in some studies. However, in this group of patients, neither sperm DNA fragmentation (Halo assay) nor oxidation (8OdHG immunodetection) measures were correlated with age by Spearman’s correlation test. When splitting the patients by age into “young” (≤35 years old) (n = 13) and “old” (>35 years old) (n = 21) groups, no statistically significant difference was found in the means of DNA fragmentation (Halo assay) or oxidation (8OdHG immunodetection) measures between groups (Supplementary Table S1). Moreover, when grouping patients into male factor or non-male-factor groups (n = 12 and n = 22, respectively), no statistically significant difference was found in the means of sperm DNA fragmentation (Halo) or oxidation (8OdHG) measures between groups (Supplementary Table S1).

Direct comparisons between this study and others prove difficult, as no universal freezing method is used across all clinics. Protocol differences include the preparation of sperm prior to freezing, the cryoprotectants used, the sperm loading and freezing devices, the speed of cooling, the timing of freezing steps and the warming method. Our group opts for a simplified semen freezing system because we found improved post-thaw motility and reduced DNA fragmentation in sperm compared to programmable slow freezing methods (unpublished observations). Furthermore, the variability between participants in our study group is an uncontrolled variable that could have affected the discordance seen in some of the results, namely, the lower-than-expected proportion of sperm with a high MMP, which is not consistent with those previously reported for similar populations [61]. To rule out a possible effect of male factor infertility, patients were divided into male factor or non-male-factor groups (n = 12 and n = 22, respectively), but no statistically significant difference was found in the means of the proportion of sperm with a high MMP (JC-1 assay) between groups (Supplementary Table S2).

## 5. Conclusions

Given the increasing demand for sperm cryopreservation and the negative impacts of semen cryopreservation on sperm health, it is imperative to protect sperm during this process. Our study highlights that mitochondrial activators—or, mitochondrial-targeted therapeutics—added to the cryopreservation medium can preserve sperm quality. One outstanding candidate, BGP-15, warrants further study since it could be beneficial in the preservation of sperm quality during the clinical preparation for assisted reproductive technologies and may raise fertilisation rates.

## Figures and Tables

**Table 1:** Demographics of the study participants.

**Number of patients**	34
**Age**, mean ± SD (range min–max)	36.7 ± 5.7 (27–51)
**BMI**, mean ± SD (range min–max)	28.9 ± 5.0 (21.3–46.1)
**Cigarette smoker**, n (%)	1 (2.9)
**Alcohol consumer**, n (%)	28 (82.4)
**Cause of infertility**	
**Male factor**, n (%)	12 (35.3)
**Female factor**, n (%)	24 (70.6)
**Unexplained**, n (%)	7 (20.6)

**Table 2:** **Mitochondrial activators affect sperm kinetics.** VCL = curve speed, VSL = linear speed, VAP = average speed, LIN = linearity index, STR = straightness index, WOB = oscillation index, ALH = amplitude of lateral head, BCF = beat cross frequency. Data represented as mean ± SD. Different letters indicate significant differences between groups.

		Fresh (n = 34)	Control (n = 34)	BGP-15 (n = 34)	MitoQ (n = 21)	L-Carnitine (n = 11)
VCL (µm s^−1^)	Mean	36.5 ± 11.4 ^a^	29.5 ± 7.7 ^b^	31.6 ± 5.9 ^b^	31.7 ± 8.1 ^b^	34.7 ± 9.0 ^a,b^
	Rapid	48.3 ± 14.7	45.3 ± 17.7	49.1 ± 13.3	46.5 ± 19.8	54.1 ± 6.8
	Medium	19.4 ± 2.1 ^a^	18.1 ± 1.7 ^b^	18.3 ± 1.4 ^b^	18.4 ± 2.0 ^b^	19.1 ± 1.7 ^a,b^
	Slow	19.4 ± 2.1 ^a^	18.1 ± 1.7 ^b^	18.3 ± 1.4 ^b^	18.4 ± 2.0 ^b^	19.1 ± 1.7 ^a,b^
VSL (µm s^−1^)	Mean	13.0 ± 5.1 ^a^	8.6 ± 3.4 ^b^	9.2 ± 2.8 ^b^	9.3 ± 3.3 ^b^	10.7 ± 3.2 ^a,b^
	Rapid	27.3 ± 8.4 ^a^	23.6 ± 9.7 ^b^	25.9 ± 7.8 ^a,b^	24.9 ±10.6 ^a,b^	28.0 ± 2.9 ^a,b^
VAP (%)	Mean	20.84 ± 6.9 ^a^	4.8 ± 1.2 ^b^	10.5 ± 1.9 ^b^	23.5 ± 4.2 ^b^	60.2 ± 6.8 ^a,b^
	Rapid	31.2 ± 9.4 ^a^	27.0 ± 11.1 ^b^	29.6 ± 8.7 ^a,b^	28.7 ±12.1 ^a,b^	32.1 ± 3.1 ^a,b^
LIN (%)	Mean	32.7 ± 7.9 ^a^	26.5 ± 6.3 ^b^	27.2 ± 5.0 ^b^	27.6 ± 5.3 ^b^	28.8 ± 4.9 ^a,b^
	Rapid	54.5 ± 15.7 ^a^	46.9 ± 19.3 ^b^	50.9 ±15.2 ^a,b^	47.7 ±19.8 ^a,b^	53.4 ± 3.5 ^a,b^
STR (%)	Mean	55.1 ± 8.4 ^a^	46.3 ± 10.3 ^b^	49.9 ± 6.2 ^b^	49.3 ± 6.5 ^b^	51.5 ± 5.3 ^b^
	Rapid	82.3 ± 21.0	77.4 ± 28.8	82.0 ± 20.9	74.9 ± 29.9	87.2 ± 1.2
WOB (%)	Mean	56.0 ± 7.1 ^a^	50.2 ± 6.7 ^b^	50.5 ± 4.7 ^b^	51.9 ± 4.4 ^b^	52.7 ± 5.3 ^a,b^
	Rapid	62.0 ± 17.5 ^a^	53.5 ± 22.0 ^b^	57.9 ± 16.7 ^a,b^	54.7 ± 22.5 ^a,b^	61.1 ± 3.7 ^a,b^
ALH (µm)	Mean	2.00 ± 0.48 ^a^	1.72 ± 0.34 ^a^	1.82 ± 0.30 ^a^	1.84 ± 0.36 ^a^	1.93 ± 0.37 ^b^
	Rapid	2.35 ± 0.58	2.21 ± 0.88	2.38 ± 0.65	2.32 ± 1.08	2.52 ± 0.29
BCF (Hz)	Mean	4.64 ± 1.52 ^a^	2.95 ± 1.15 ^b^	3.27 ± 1.05 ^c^	3.18 ± 1.33 ^b^	3.87 ± 1.04 ^d^
	Rapid	7.52 ± 2.12 ^a^	6.08 ± 3.13 ^b^	6.47 ± 2.76 ^b^	6.20 ± 3.11 ^b^	6.85 ± 1.58 ^b^

## Data Availability

Data are contained within the article.

## Supplementary Materials

The following supporting information can be downloaded at: https://www.mdpi.com/article/10.3390/antiox11091808/s1, Table S1: Subgroup analyses for age effects on DNA damage; Table S2: Subgroup analyses for male factor infertility effect on DNA damage and the percentage of high mitochondrial membrane potential.
