# Supplementary material for: Restoring Sperm Quality Post-Cryopreservation Using Mitochondrial-Targeted Compounds
Source: Antioxidants (Basel). 2022 Sep 14;11(9):1808. doi: 10.3390/antiox11091808 (PMC9495717; doi:10.3390/antiox11091808)
Supplement: Supplementary file 1 [file antioxidants-11-01808-s001.zip › antioxidants-1837754-supplementary.pdf]

Supplementary Table S1. Subgroup analyses for age effects on DNA damage.

**Age**	**≤35**	**>35**	**P-value***
**n** (%)	13 (38.2)	21 (61.8)	
**HALO**, mean ± SD	12.1 ± 7.6	15.8 ± 9.4	0.2
**8OHdG**, mean ± SD	12.2 ± 5.9	10.1 ± 5.1	0.3

* Unpaired, Two-tailed Student's T-test

Supplementary Table S2. Subgroup analyses for male factor infertility effect on DNA damage and percentage of high mitochondrial membrane potential.

Cause of Infertility	Non-Male Factor	Male Factor	P-value*
**n** (%)	22 (64.7)	12 (35.3)	
**HALO**, mean ± SD	13.5 ± 7.5	16.1 ± 11.0	0.4
**8OHdG**, mean ± SD	10.8 ± 5.9	11.2 ± 4.6	0.8
**High MMP %,** mean ± SD	0.3 ± 0.2	1.4 ± 0.9	0.07

* Unpaired, Two-tailed Student's T-test
